# To NFκB or not to NFκB: The Dilemma on How to Inhibit a Cancer Cell Fate Regulator

**Published:** 2012-10-11

**Authors:** Daniela Sorriento, Maddalena Illario, Rosa Finelli, Guido Iaccarino

**Affiliations:** 1 Department of Clinical Medicine, Cardiovascular and Immunological Science, Federico II University of Naples, Italy; 2 Department of Cellular and Molecular Biology and Pathology, Federico II University of Naples, Italy; 3 Department of Medicine and Surgery, University of Salerno, Italy; 4 IRCCS “Multimedica”, Milano, Italy

**Keywords:** transcription factors, IκB, GRK5, cancer

## Abstract

Nuclear factor κB (NFκB) is a transcription factor that plays an important role in carcinogenesis as well as in the regulation of inflammatory response. NFκB is constitutively expressed in tumours where it induces the expression of genes which promote cell proliferation, apoptotic events, angiogenesis, invasion and metastasis. Furthermore, many cancer cells show aberrant or constitutive NFκB activation that mediates resistance to chemo- and radio-therapy. Therefore, the inhibition of NFκB activity appears a potential therapeutic strategy for cancer treatment. In this review, we focus on the role of NFκB in carcinogenesis and summarize actual inhibitors of NFκB that could be potential therapeutic target in cancer therapy.

## I. INTRODUCTION

Human cancer is a complex disease based on multiple etiologies, multiple cell targets, and distinct developmental stages. Cancer cells share common features that regulate cell proliferation and homeostasis [1] including resistance to growth inhibitory signals, self-sufficiency in growth, resistance to apoptosis, extended replication potential, enhanced angiogenic potential, and the ability to invade local tissue and to metastasize to distant sites [1]. Autonomous cell growth characterizes cancer cells and depends on impaired expression of growth factors or growth factor receptors, leading to uncontrolled cell proliferation. Thus, a fairly common mechanism in cancer is the up-regulation of expression of members of the epidermal growth factor receptor family such as EGF receptor or Her2/ErbB2. Furthermore, certain cancer cells produce growth factors such as PDGF and TGF-α, which can promote cell proliferation in an autocrine manner [1,2]. Mutations in proteins that regulate cell proliferation are also relatively common in cancer. For example, resistance to growth inhibitory signals are due to mutations in tumour suppressor genes such as p53, Rb, Arf, and APC, or in receptors such as those for TGFβ. Additionally, up-regulation of expression of cyclin D1 or c-myc, or activating mutations in transcription factors can promote cell proliferation or cell growth [1,2]. A key process in the ability of tumour cells to spread is the suppression of apoptotic potential. Resistance to apoptosis can involve the activation of expression of anti-apoptotic factors, such as Bcl-2 or Bcl-xL, or the loss of expression or mutation of pro-apoptotic factors, such as p53 [2]. Additionally, mutation in tumour suppressors such as PTEN leads to the activation of intracellular signalling pathways (in this case, the PI3 kinase/Akt pathway) that suppress apoptosis [3]. An additional mechanism of suppression of cancer cell apoptosis can be derived from release of cytokines from the tumour stroma [2]. The ability of cancer cells to metastasize depends on angiogenesis which in turn is mediated through a complex interplay of regulatory factors, including vascular endothelial growth factor (VEGF). In fact, many tumors exhibit up-regulation of VEGF [1]. Local invasion is mediated by changes in expression of cell adhesion molecules and integrins, and in changes in expression of extracellular proteases such as MMP-2 and MMP-9. In some situations, the matrix-degrading proteases are produced by the tumour-associated stromal and inflammatory cells [2].

## II. TRANSCRIPTION FACTORS AND NFκB

Transcription factors are gene regulatory proteins that bind to the promoter or enhancer regions of target genes and induce either transcriptional repression or activation [4]. The basic structure of a transcription factor mainly contains a DNA-binding domain and an activator domain. DNA-binding motifs include zinc-finger, helix-loop-helix, helix-turn-helix, leucine zipper and high-mobility groups, based on which transcription factors are classified [4,5]. The activator domain of these transcription factors interacts with components of transcription machinery such as RNA polymerases and associated transcription regulators. Transcription factors regulate gene expression in different ways: they stabilize or block the binding of RNA polymerase to DNA; catalyse the acetylation or deacetylation of histone proteins; recruit co-activator or co-repressor proteins to the transcription factor DNA complex [4,6,7]. Transcription factors represent prime targets for disruption in many diseases [8]. In cancer, for instance, a number of oncogenic transcription factors such as activator protein 1 (AP-1), nuclear factor κB (NFκB), and signal transducer and activator of transcription (STAT)-3/STAT5 are constitutively expressed and thus may present promising targets for cancer prevention [9]. Among them, NFκB is an ubiquitously expressed and highly regulated dimeric transcription factor that regulates the expression of genes responsible for innate and adaptive immunity, tissue regeneration, stress responses, apoptosis, cell proliferation, and differentiation [10]. NFκB has now been shown to contribute to the pathogenesis of a large number of diseases including cancer, diabetes, allergy, rheumatoid arthritis, Crohn’s disease, cardiovascular diseases, atherosclerosis, Alzheimer’s disease, muscular dystrophy, cardiac hypertrophy, catabolic disorders, hypercholesterolemia, ischemia/reperfusion [10].

## III. NFκB SIGNALLING PATHWAY

**Fig 1:**
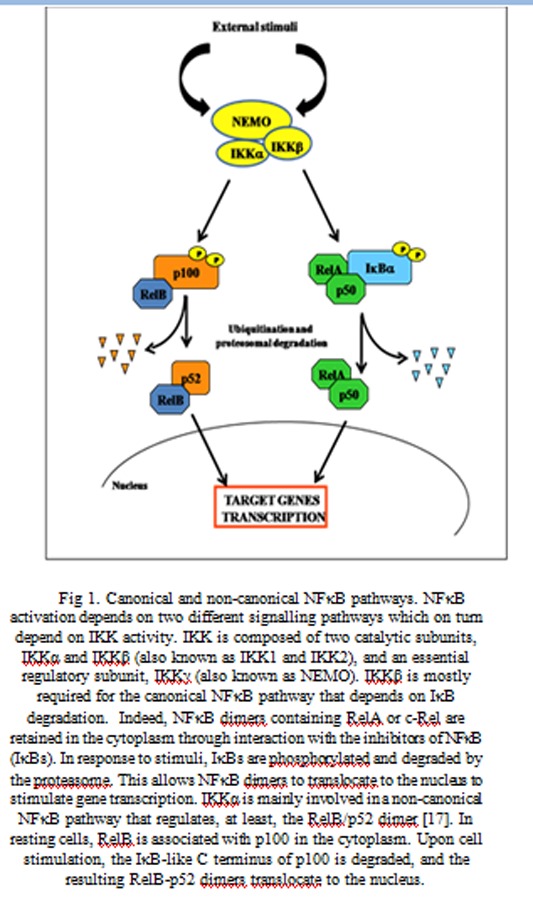
Canonical and non-canonical NFκB pathways. NFκB activation depends on two different signalling pathways which on turn depend on IKK activity. IKK is composed of two catalytic subunits, IKKα and IKKβ (also known as IKK1 and IKK2), and an essential regulatory subunit, IKKγ (also known as NEMO). IKKβ is mostly required for the canonical NFκB pathway that depends on IκB degradation. Indeed, NFκB dimers containing RelA or c-Rel are retained in the cytoplasm through interaction with the inhibitors of NFκB (IκBs). In response to stimuli, IκBs are phosphorylated and degraded by the proteasome. This allows NFκB dimers to translocate to the nucleus to stimulate gene transcription. IKKα is mainly involved in a non-canonical NFκB pathway that regulates, at least, the RelB/p52 dimer [17]. In resting cells, RelB is associated with p100 in the cytoplasm. Upon cell stimulation, the IκB-like C terminus of p100 is degraded, and the resulting RelB-p52 dimers translocate to the nucleus.

NFκB belongs to the Rel family, comprising the following proteins: RelA (p65), c-Rel, RelB, NFκB 1 (p50/p105) and NFκB 2 (p52/100) [10,11]. While RelA, c-Rel and RelB are synthesized as final proteins, p50 and p52 derive from large precursors p105 and p100, respectively, after processing by the proteasome. The nuclear activity of NFκB is controlled by shuttling from the cytoplasm to the nucleus in response to cell stimulation. It has been demonstrated that NFκB activation depends on two different signalling pathways, which can be referred to as canonical and non-canonical pathway [12]. In the canonical pathway, NFκB dimers containing RelA or c-Rel are retained in the cytoplasm through interaction with the inhibitors of NFκB (IκBs). In response to a variety of stimuli, IκBs are phosphorylated (Ser32 and Ser36 for IκBα and Ser19 and Ser21 for IκBβ) by the activated IκB kinase (IKK) complex, followed by rapid ubiquitin-dependent degradation by the proteasome [12,13]. This allows NFκB dimers to translocate to the nucleus, where they stimulate the expression of target genes. IKK is composed of two catalytic subunits, IKKα and IKKβ (also known as IKK1 and IKK2), and an essential regulatory subunit, IKKγ (also known as NEMO) [12]. While IKKβ is mostly required for the canonical NFκB pathway that depends on IκB degradation [14–16], IKKα is involved in a non-canonical NFκB pathway that regulates, at least, the RelB/p52 dimer [17]. In resting cells, RelB is associated with p100 in the cytoplasm. Upon cell stimulation, the IκB-like C terminus of p100 is degraded, and the resulting RelB-p52 dimers translocate to the nucleus [18] (fig 1).

## IV. NFκB ACTIVATION IN CANCER

**Fig. 2:**
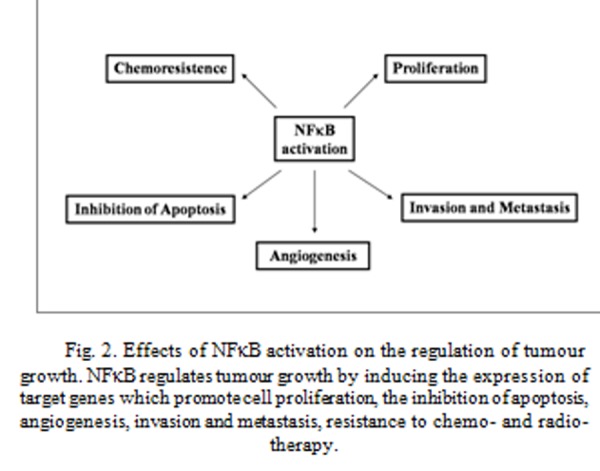
Effects of NFκB activation on the regulation of tumour growth. NFκB regulates tumour growth by inducing the expression of target genes which promote cell proliferation, the inhibition of apoptosis, angiogenesis, invasion and metastasis, resistance to chemo- and radio-therapy.

Strong evidences suggest a key role of NFκB in cancer. According to Hanahan and Weinberg, the tumour genesis requires six essential alterations to normal cell physiology: self-sufficiency in growth signals; insensitivity to growth inhibition; evasion of apoptosis; immortalization; sustained angiogenesis; and tissue invasion and metastasis [1]. NFκB is able to regulate several of these cellular alterations (fig 2), and has been shown to be constitutively activated in some types of cancer cell [2,19,20].

This phenomenon seems to be dependent on several mechanisms in different cancers: aberrant IKK activity, a shorter half-life of IκB in B-cell lymphoma, mutated IκB in Hodgkin’s lymphoma, IL-1β production in acute myelogenous leukaemia, TNF production in cutaneous T-cell lymphoma and Burkitt’s lymphoma [21,22]. It has been shown that the avian REV-T oncovirus produces the constitutively active v-REL oncoprotein, which causes rapidly progressing lymphomas and leukaemias [19,23]. The TAX oncoprotein of human T-cell leukaemia virus (HTLV)-1 has been shown to directly interact with the IKK complex, inducing its constitutively activation which results in the activation of both NFκB signalling pathways [19,24]. Other viral oncoproteins have also been shown to activate NFκB by means of different mechanisms [25]. Moreover, chromosomal amplification, rearrangement and other genetic aberrations of genes coding for NFκB family members are present in many solid tumours and cause NFκB activation [20]. Indeed, cancer-associated genetic modifications of genes encoding for NFκB and IκB proteins induce uncoupling of NFκB factors from their regulators, causing constitutive NFκB activation [19]. Finally, autocrine and paracrine production of pro-inflammatory cytokines, oncogenic activation of upstream signalling molecules and chronic infections have been shown to persistently stimulate IKK activity, which leads to constitutive NFκB activation [19].

## V. EFFECTS OF NFκB ACTIVATION ON TUMOUR GROWTH

NFκB activation regulates tumour genesis by inducing the expression of target genes which promote cell proliferation, inhibition of apoptosis, angiogenesis, invasion and metastasis, resistance to chemo- and radio-therapy (fig 2).

### A. NFκB and apoptosis

It has been demonstrated that NFκB exerts a dual function on apoptosis, either as an inhibitor or an activator, depending on stimuli, cell type and subunit involved [26–29].

For instance, it is generally accepted that NFκB activation is responsible of induction of apoptosis in cardiac cells [28] and apoptosis resistance in cancer cells [27]. This latter event occurs by inducing the expression of multiple anti-apoptotic proteins and interfering with the expression or activity of pro-apoptotic proteins. Indeed, NFκB may activate the transcription of several genes involved in the suppression of cell death by both mitochondrial (intrinsic) and death receptor (extrinsic) pathways [30]. The release of cytochrome c from mitochondria directly triggers caspase-3 activation through formation of the cytochrome c/Apaf-1/caspase-9-containing apoptosome complex [31]. It has been demonstrated that NFκB activation suppresses mitochondrial release of cytochrome c through the activation of the Bcl-2 family member A1/Bfl-1 [32]. NFκB may up-regulate the expression of proteins that interfere with the death receptor apoptotic pathway such as the FLICE-like inhibitory protein (FLIP) [33,34]. FLIP competes with caspase-8 for the binding to the Death-Inducing Signalling Complex (DISC). Thus, high levels of FLIP prevent caspase-8 recruitment to the DISC. It has been reported an up-regulation of FLIP in many tumours which could explain the resistance to death receptor apoptosis [35–39]. Other proteins, TRAF2 and TRAF6, activated by TNF may also be targets of NFκB and may lead to activation of pro-survival signalling [40]. NFκB also induces the expression of the Inhibitors of Apoptosis (IAPs) [41,42] and some members of the anti-apoptotic Bcl-2 family [43,44]. The IAPs (c-IAP1, c-IAP2, and XIAP) suppress apoptosis through direct inhibition of effector caspases (caspases-3, -6, -7, and 9) [40,45], while the anti-apoptotic members of the bcl-2 family antagonize the function of the pro-apoptotic members A1/BFL1 and Bcl-XL [46].

Furthermore, NFκB may interfere with the transcriptional activity of p53. In healthy cells, the level of p53 remains typically low under the control of Mdm2, which is responsible for p53 ubiquitination leading to its rapid degradation [47]. In turn, synthesis of Mdm2 transcript is controlled by p53 [48], which defines the negative feedback. DNA damage activates the checkpoint proteins, which destabilize Mdm2 and trigger p53 phosphorylation elevating its stability and transcriptional activity [49]. This disturbs the homeostatic balance between Mdm2 and p53 leading to oscillations and/or rise of the p53 level. Activated p53 triggers transcription of groups of genes, products of which are responsible for cell cycle arrest and DNA repair and, if the last fails or takes too long, for initiation of apoptosis. In tumour cells, NFκB inhibits p53-induced apoptosis, by up-regulating anti-apoptotic genes, and down-regulating p53 levels.

### B. NFκB and proliferation

Several genes, such as TNF, IL-1β and IL-6, that mediate cell proliferation are under the transcriptional control of NFκB. Besides these growth factors, certain cell cycle regulatory proteins are also regulated by NFκB. In particular, NFκB promotes cell cycle progression, by regulating the expression of cyclins D1, D2, D3, cyclin E 50–53 and c-myc [54–56]. NFκB-induced cyclin D1 expression appears to be a key element in mammary gland development and breast carcinogenesis [57]. It was shown that growth factors like epithelial growth factor and platelet-derived growth factor induce proliferation of tumour cells through activation of NFκB [58]. It has been reported that proliferation of Hodgkin/Reed-Sternberg cells depends on activated NFκB [59,60]. As happens in apoptosis, NFκB exert a reciprocal regulation of cell proliferation by inducing inhibition or stimulation, depending on cell type. For example, NFκB activation can suppress the proliferation of keratinocytes 61 and c-Rel overexpression induces cell cycle arrest in HeLa cells 62. On the other hand, NFκB induces the expression of cell adhesion molecules (ICAM-1, E-selectin), and proteins involved in invasion (matrix metallo-proteinases). However, generally, in tumour cells NFκB induces cell proliferation and the expression of angiogenic factors.

### C. NFκB and angiogenesis

Metastasis of cancer cells is a complex process involving multiple steps, including invasion, angiogenesis, trafficking of cancer cells through blood vessels, extravasations, organ specific homing, and growth. Proteins like matrix metalloproteinase 2 (MMP2), MMP9 and serine protease urokinase-type plasminogen activator (uPA) which play an important role in tumour invasion and metastasis, are under the transcription control of NFκB. Indeed, it has been demonstrated that NFκB blockade induces down-regulation of pro-metastatic MMP-9, uPA, and heparanase and reciprocal up-regulation of anti-metastatic TIMP-1 and -2 and PAI 2 [63]. Furthermore, NFκB regulates the expression of intracellular adhesion molecule 1 and vascular cell adhesion molecule 1 that are associated with tumour metastasis [64]. Tumour angiogenesis is regulated by chemokines (monocyte chemo-attractant protein-1, IL-8) and growth factors (TNF, VEGF) produced by macrophages, neutrophils and other inflammatory cells [65]. The production of these angiogenic factors has been shown to be regulated by NFκB activation [66,67]. It has been demonstrated that NFκB promotes breast cancer cell migration and metastasis by inducing the expression of the chemokine receptor CXCR4 [68]. Huang et al reported that blockade of NFκB signalling also inhibits angiogenesis of human ovarian cancer cells by suppressing expression of VEGF and IL-8 [69]. Cyclooxygenase 2, which is up-regulated in more aggressive forms of colorectal cancer, is known to be transcriptionally activated by NFκB and promote angiogenesis [70].

### D. NFκB and chemo-resistance

Tumours with constitutive NFκB activation usually show increased resistance to chemotherapy [71]. It has been suggested that NFκB may induce the expression of the multidrug resistance P-glycoprotein, involved in the development of resistance to chemotherapy drugs in many cancers [72]. In some tumours, cells exposed to radiation or certain chemotherapeutic drugs show enhanced activation of NFκB [71]. On the other hand, inhibition of NFκB improves the apoptotic response to radiation therapy [71,73]. For instance, it has been found that inhibition of NFκB activation confers sensitivity to TNF-α by impairment of cell cycle progression in six human malignant glioma cell lines [73]. Inhibitors of NFκB activation can block the neoplastic transformation response. Indeed, inhibition of NFκB through adenoviral delivery of a modified form of IκB, a specific inhibitor of NFκB, has been reported to sensitize chemo-resistant tumours to the apoptotic potential of TNF-α and to the chemotherapeutic compound CPT-11, resulting in tumour regression [74].

## VI. INFLAMMATION AND CANCER

A link between inflammation and cancer has been suspected for many years. While acute inflammation is a part of the defence response, chronic inflammation can mediate several diseases, including cardiovascular diseases, cancer, diabetes, arthritis, autoimmune diseases [75]. Since NFκB becomes activated in response to inflammatory stimuli and its constitutive activation has been associated with cancer, NFκB represents the link between these two processes. Indeed, several pro-inflammatory gene products have been associated to tumour genesis and they are all under the transcription control of NFκB. In particular, TNF, interleukins, chemokines, COX-2, 5-LOX, and MMP-9 have all a key role in cancer development [75,76].

### A. Role of cytokines in cancer

Several inflammatory interleukins, including IL-1, IL-6, IL-8, and IL-18, are associated to tumour genesis. Secretion of IL-1α promotes growth of cervical carcinoma [77] while autocrine production of interleukin IL-1β promotes growth and confers chemoresistance in pancreatic carcinoma cell lines [78]. IL-1β secretion into the tumour milieu also induces several angiogenic factors from tumour and stromal cells that promotes tumour growth through an increase of neovascularization in lung carcinoma growth in vivo [79]. IL-6 acts as a paracrine growth factor for multiple myeloma, non-Hodgkin’s lymphoma, bladder cancer, colorectal cancer, and renal cell carcinoma (RCC) [80–84]. Several evidences underline the key role of TNF-α as mediator of inflammation and cancer [85,86]. Although initially thought to be a product only of macrophages, many malignant tumours are characterized by a constitutive production of TNF-α from the tumour microenvironment and its presence often associates with poor prognosis. Indeed, TNF-α is produced by a wide variety of tumour cells, including those of B cell lymphoma [87], cutaneous T cell lymphoma [88], megakaryoblastic leukaemia [89], adult T cell leukaemia [90], AML [91], CLL [92],ALL [93], breast carcinoma [94], colon carcinoma, lung carcinoma, squamous cell carcinoma, pancreatic cancer [95], ovarian carcinoma [96]. As TNF-α receptors are expressed on both epithelial and stromal cells, TNF-α can directly facilitate cancer development by regulating the proliferation and survival of neoplastic cells; alternatively it can also act on endothelial cells and other inflammatory cells present at the tumour microenvironment [97]. Tumour stromal cells, including macrophages, dendritic cells and fibroblasts, release several inflammatory cytokines, such as TNF-α, IL-1 and IL-6, which attract and recruit more inflammatory cells to the tumour microenvironment to further enhance the proliferation and survival of tumour cells. TNF-α is involved in all steps of tumour genesis, including cellular transformation, promotion, survival, proliferation, invasion, angiogenesis, and metastasis. Indeed, several reports indicate that TNF-α induces cellular transformation, proliferation, and tumour promotion [86,98–101]. First, TNF-α induces tumour initiation and promotion and enhances tumour cell proliferation. All these action are mediated by the activation of NFκB. Indeed, in mouse epidermal JB6 cells, TNF-α treatment increases NFκB activity in a dose dependent manner and TNF-α induced NFκB activation is essential for neoplastic transformation of these cells [102]. TNF-α also promotes tumour cell survival by inducing genes coding for NFκB dependent anti-apoptotic molecules [103]. In addition TNF-α not only acts as an autocrine growth factor but also induces the expression of other growth factors such as amphiregulin, EGFR and TGF-α, leading to increased tumour proliferation. For instance, in cervical cells TNF-α induces amphiregulin, which stimulates cell proliferation [77], whereas in pancreatic cells TNF-α induces the expression of epidermal growth factor receptor (EGFR) and transforming growth factor (TGF-α), which mediates proliferation. Finally TNF-α enhances tumour angiogenesis through different angiogenic factors such as IL-8 and VEGF, and also is a critical regulator of VEGF and jagged-1 expression via a JNK- and AP-1- dependent pathway [104].

It has been demonstrated that tumour necrosis factor (TNF-α) has a therapeutic role when expressed locally by the cells of the immune system [105]. The anti-cancer actions of TNF can be due to direct effects on tumour cells and/or indirect effects on host stroma, and many of these effects are potentiated by IFN-γ. Vascular damage is widely accepted as a mechanism of its anti-tumour effects. In a breast cancer xenograft model, locally injected human TNF resulted in growth inhibition of established tumours. However macroscopic necrosis was observed in these mice when systemic rat IFN-γ, which has no activity alone, was also given. Within 4 h of administration of this cytokine combination, platelet adherence to tumour cells was observed, followed by destruction of the tumour vasculature. Both necrosis and apoptosis of tumour cells was demonstrated and there was up-regulation of mRNA for a range of stromal cytokines and adhesion molecules [106].

TNF-α produced by tumours can act as an endogenous tumour promoter [98]. Komori’s group reported that human TNF-α is 1000 times more effective than the chemical tumour promoters okadaic acid and 12-O-tetradecanoylphorbol-13-acetate in inducing cancer [107]. In most of these cells, TNF-α acts as an autocrine growth factor, however in some cell types TNF-α induces the expression of other growth factors, which mediate proliferation of tumours. TNF-α has been reported to induce angiogenic factor up-regulation in malignant glioma cells [108] which in turn promotes angiogenesis and tumour progression. TNF-α could enhance invasiveness of some carcinomas or stimulate epithelial wound healing in vivo [109] and it has been even reported to mediate macrophage-induced angiogenesis [110].

### B. Role of chemokines in cancer

The chemokines are soluble, small proteins that bind to their associate G-protein coupled receptors (GPCRs) to elicit a cellular response [111]. Tumour cells secrete and respond to chemokines, which in turn facilitate cancer growth through means of increased angiogenesis, inflammation, endothelial cell recruitment and cell migration. Furthermore, chemokines regulate the recruitment and trafficking of leukocytes to sites of inflammation. Chemokines are grouped into four classes based on the positions of key cysteine residues: C, CC, CXC, and CX3C. Different classes of chemokines recognize different subset of cells, expressing the corresponding receptor [112] (Table 1).

Tumour cells also release soluble mediators such as VEGF-A (vascular endothelial growth factor-A), TGF-β and TNF-α that act on myeloid and endothelial cells and induce the expression of non-classical chemokines such as the S100 chemokine. Interestingly, S100 chemokines are implicated in targeting of the tumour cells to the pre-metastatic sites rather than the metastatic sites [113,114]. Evidence from murine models and human cancers suggests that CC chemokines are major determinants of macrophage and lymphocyte infiltration in melanoma, ovarian carcinoma, breast, and cervix, and in sarcomas and gliomas [115]. The chemokines elaborated from the tumour and the stromal cells bind to the chemokine receptors present on these cells. The two chemokine receptor–chemokine pairs that are involved commonly in many tumour are CXCR4–CXCL12 and CCR7–CCL21 [112]. Chemokine receptors CXCR4 and CCR7 are highly expressed in human breast cancer cells, malignant breast tumours, and metastasis [116]. Their respective ligands CXCL12/ SDF-1a and CCL21/6Ckine are highly expressed in organs representing the first destinations of breast cancer metastasis. In breast cancer cells, signalling through CXCR4 or CCR7 mediates actin polymerization and pseudopodia formation and subsequently induces chemotactic and invasive responses. In vivo, neutralizing the interactions of CXCL12/CXCR4 significantly impairs metastasis of breast cancer cells to regional lymph nodes and lung. Malignant melanomas show high expression levels of CCR10 in addition to CXCR4 and CCR7. Thus chemokines and their receptors have a critical role in determining the metastatic destination of tumour cells. Melanoma growth stimulatory activity/growth-regulated protein (MGSA/GRO), is a CXC chemokine constitutively expressed in melanoma tumours and is associated with constitutive NFκB activity [117]. Ovarian cancers express CXCR4 chemokine receptors [118]. Its ligand, CXCL12 (stromal cell-derived factor 1), in ovarian cancer cells stimulates cell migration and invasion through extracellular matrix, as well as DNA synthesis and EGFR transactivation [119,120].

### C. Role of matrix metallo-proteinases in cancer

NFκB regulates several dependent-matrix metallo-proteinases (MMPs), which are correlated with malignant prognosis of various cancer types including colorectal, breast, and bladder cancers [121]. Indeed, the analyses on the human MMP-9 gene promoter revealed that NFκB is one of major transcription factors responsible for its induction [121]. MMPs are key modulators of many biological processes during pathophysiological events, such as skeletal formation, angiogenesis, cellular migration, inflammation, wound healing, and cancer [122]. By means of in vivo selection, transcriptomic analysis, functional verification and clinical validation, Minn et al have identified a set of genes comprising MMPs, that marks and mediates breast cancer metastasis to the lungs. In particular, MMP-2 acts mainly as virulence gene that may allow tumours to aggressively invade, colonize and grow in the lungs without markedly contributing to primary tumour growth, whereas MMP-1, determine metastatic potential of breast cancer to produce lung metastases [123]. MMP-7 also promotes cancer invasion by proteolytic cleavage of the extracellular matrix substrates and activates other MMPs, such as proMMP-2 and proMMP-9, to facilitate tumour invasion [124]. It has been demonstrated that transgenic mice lacking MMP-9 show reduced keratinocyte hyper proliferation at all neoplastic stages and a decreased incidence of invasive tumours [125]. Yet those carcinomas that do arise in the absence of MMP-9 show a greater loss of keratinocyte differentiation, indicative of a more aggressive and higher grade tumour [125].

## VII. NFκB INHIBITION AND CANCER THERAPHY

It is known that a sustained, constitutive activation of NFκB contributes to malignant progression and therapeutic resistance in most of the major forms of human cancer, such as human lymphomas [60], carcinomas of the breast [126], prostate [127], lung [128], colon [129], pancreas [130], thyroid [131], head and neck [132] and cervix [133]. Thus, the modulation of NFκB activity could represent an useful therapeutic strategy for cancer, since NFκB inhibition promotes apoptotic events induced by chemotherapy, reduces the high proliferative rate that characterizes tumour cells and inhibits metastasis [134]. To date, different approaches have been developed to block NFκB in several conditions by regulating different steps in NFκB signalling pathway:

### A. IKK inhibition

A protein that disrupts the association of the IKK complex is used to prevent inflammatory bone destruction [135]. Similarly, the inhibition of IκBα phosphorylation by the Bay 11-7082 compound, has been successfully used to prevent tumour growth and leukemic infiltration in a mouse model of adult T cell leukaemia [136]. The IKK inhibitors BAY 11-7082 and BAY 11-7085 also induce the apoptosis of colon cancer cells [137]. Some anti-inflammatory drugs and other substances such as curcumin, trans-resveratrol or parthenolide may inhibit NFκB by interfering with IKK activity [138–142]. Curcumin is a polyphenol derived from the plant Curcuma longa that exerts anti-oxidant, anti-inflammatory, anti-angiogenic and anti-tumoral activity. It was found to suppress COX-2 expression by inhibiting extracellular signal-regulated kinase (ERK) activity and NFκB in phorbol ester-induced mouse skin tumour genesis [143]. Non-steroidal anti-inflammatory agents (NSAIDs), including aspirin, have been shown to suppress NFκB activation by inhibiting IKK activation and IκBα degradation in tumour cells [144]. A small molecule inhibitor of IKK (PS-1145) was found to be selectively toxic for subtypes of diffuse large B-cell lymphoma cells that are associated with NFκB activation [2,145]. This compound was shown to lead to down-regulation of a set of NFκB-dependent genes [2].

### B. IκB upregulation

**Fig 3:**
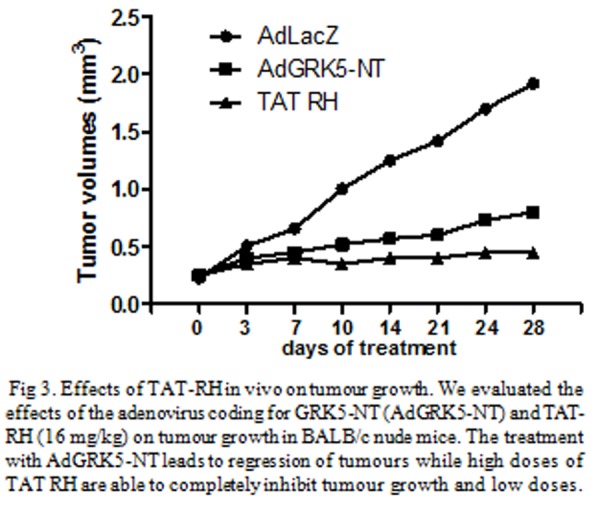
Effects of TAT-RH in vivo on tumour growth. We evaluated the effects of the adenovirus coding for GRK5-NT (AdGRK5-NT) and TAT-RH (16 mg/kg) on tumour growth in BALB/c nude mice. The treatment with AdGRK5-NT leads to regression of tumours while high doses of TAT RH are able to completely inhibit tumour growth and low doses.

The inhibition of NFκB activation by expression of a degradation, increased NFκB dependent apoptosis to stimuli such as TNFα [146]. Zhou et al, transfected the dominant-negative mutant inhibitor of NFκB (IκBm) into an acute lymphoblastic leukaemia (ALL) cell line with constitutive NFκB activation [147]. Overexpression of IκBm simultaneously down-regulates NFκB activation and sequesters p53 in the cytoplasm, thus enhancing NFκB-regulated apoptosis but blocking p53-dependent apoptosis [147]. We have demonstrated that in vitro, adenovirus mediated overexpression of the RH domain of GRK5 (AdGRK5-NT) in human tumour cells (KAT-4) induces IκB accumulation and inhibits NFκB transcriptional activity leading to apoptotic events 27. In BALB/c nude mice harbouring KAT-4 induced neoplasias, intra-tumour delivery of AdGRK5-NT reduces in a dose-dependent fashion tumour growth, with the highest doses completely inhibiting it. This phenomenon is paralleled by a decrease of NFκB activity, an increase of IκB levels and apoptotic events [27]. To move towards a pharmacological setup, we synthesized the TAT-RH protein. In cultured KAT-4 cells, different dosages of TAT-RH reduced cell survival and increased apoptosis. In BALB/c mice, the anti-proliferative effects of TAT-RH appear to be dose-dependent and highest dose completely inhibits tumor growth [27] (fig 3).

### C. Proteasome inhibition

Another way to approach NFκB inhibition is to target the process of proteasome degradation. Proteasome inhibitors prevent NFκB activation by blocking the degradation of IκBs, NFκB1/p105 or NFκB2/p100. A successful strategy is using a proteasome inhibitor, Bortezomib or PS-341, to treat patients with refractory or resistant multiple myeloma [148]. Bortezomib is a dipeptidyl boronic acid that specifically inhibits 26S proteasome, the principal regulator of intracellular protein degradation like IκB. The treatment with this compound alone or in combination with other drugs, inhibits proliferation and induces apoptosis in several solid tumours [149–153], and is currently approved for treatment of multiple myeloma [152]. The importance of NFκB in multiple myeloma is suggested from its involvement downstream of CD40, the TNF receptor family member that is expressed in a variety of B-cell malignancies and which is associated with multiple myeloma homing. Consistent with this, monoclonal antibodies to CD40 block CD40L-induced NFκB activation as well as IL-6 and VEGF secretion in cultures of multiple myeloma cells and bone marrow-derived stromal cells. Other haematological malignancies are susceptible to NFκB inhibition. Proteasome inhibition blocks cell growth and induces apoptosis in adult T-cell leukaemia, an NFκB-relevant malignancy, correlated with stabilized IκBα and inhibited NFκB [2,153].

All these approaches open new fields for the management of NFκB-associated diseases like cancer. Clinical trials are being performed with some of the above described drugs and more other compounds that are able to block NFκB activity but the most significant clinical data comes from studies with the protease inhibitor bortezomib.

## VIII. CONCLUSIONS AND FUTURE PROSPECTS

It is now well established that NFκB has a key role in carcinogenesis and that the inhibition of NFκB is a promising strategy for cancer therapy. Therefore, an increasing number of compounds able to block NFκB activity at different stages of its signalling pathway have been tested. Most of these drugs have given promising results in preclinical models of tumour (pancreas, lung, colon, ovarian and breast cancer), but failed in the clinical efficacy. Actually, the only pharmacological inhibitors of NFκB approved for clinical use are proteasome inhibitors for treatment of multiple myeloma or adult T-cell leukaemia, for whose pathogenesis it has been clearly demonstrated the key role of NFκB. The difficulty to find an efficient drug for cancer treatment is due to the fact that these drugs are able to block not only the oncogenic activity of NFκB but also its physiological roles in immunity, inflammation and cellular homeostasis. Moreover, the treatment is not specifically targeted on tumour cells thus affecting also healthy cells. Finally, these drugs induce many highly toxic side effects. In the future, new drugs might be designed that should be more specific in their function, in order to avoid affecting the induction of genes that are required for immunity, and in cell targeting, in order to protect normal cells from death.

## Figures and Tables

**Table 1:** List of chemokines subfamilies and their specific target cell types

**CHEMOKINES SUBTYPE**	**TARGET CELL TYPES**
**C**	B cells, T cells, natural killer cells, neutrophils
**CC**	Dendritic cells, lymphocytes, macrophages, eosinophils, natural killer cells
**CXC**	Neutrophils, lymphocytes, endothelial and epithelial cells
**CX_3_C**	Effector T cells
